# Development of a population pharmacokinetic model of pyrazinamide to guide personalized therapy: impacts of geriatric and diabetes mellitus on clearance

**DOI:** 10.3389/fphar.2023.1116226

**Published:** 2023-05-26

**Authors:** Ryunha Kim, Rannissa Puspita Jayanti, Hongyeul Lee, Hyun-Kuk Kim, Jiyeon Kang, I-Nae Park, Jehun Kim, Jee Youn Oh, Hyung Woo Kim, Heayon Lee, Jong-Lyul Ghim, Sangzin Ahn, Nguyen Phuoc Long, Yong-Soon Cho, Jae-Gook Shin, On behalf of the cPMTb

**Affiliations:** ^1^ Center for Personalized Precision Medicine of Tuberculosis, Inje University College of Medicine, Busan, Republic of Korea; ^2^ Department of Pharmacology and PharmacoGenomics Research Center, Inje University College of Medicine, Busan, Republic of Korea; ^3^ Division of Pulmonary, Critical Care Medicine, Department of Internal Medicine, Inje University College of Medicine, Busan Paik Hospital, Busan, Republic of Korea; ^4^ Division of Pulmonology, Department of Internal Medicine, Inje University Haeundae Paik Hospital, Busan, Republic of Korea; ^5^ Division of Pulmonary and Critical Care Medicine, Department of Internal Medicine, Inje University Ilsan Paik Hospital, Goyang-si, Republic of Korea; ^6^ Department of Internal Medicine, Inje University Seoul Paik Hospital, Inje University College of Medicine, Seoul, Republic of Korea; ^7^ Pulmonary Division, Department of IM, Kosin University Gospel Hospital, Busan, Republic of Korea; ^8^ Division of Pulmonology, Department of Internal Medicine, Korea University Guro Hospital, Seoul, Republic of Korea; ^9^ Division of Pulmonary and Critical Care Medicine, Department of Internal Medicine, Incheon St. Mary’s Hospital, College of Medicine, The Catholic University of Korea, Incheon, Republic of Korea; ^10^ Division of Pulmonary, Critical Care and Sleep Medicine, Department of Internal Medicine, Eunpyeong St. Mary’s Hospital, College of Medicine, The Catholic University of Korea, Seoul, Republic of Korea; ^11^ Department of Clinical Pharmacology, Inje University Busan Paik Hospital, Busan, Republic of Korea

**Keywords:** pyrazinamide, tuberculosis, geriatric, diabetes mellitus, population pharmacokinetics, therapeutic drug monitoring

## Abstract

**Objectives:** This study was performed to develop a population pharmacokinetic model of pyrazinamide for Korean tuberculosis (TB) patients and to explore and identify the influence of demographic and clinical factors, especially geriatric diabetes mellitus (DM), on the pharmacokinetics (PK) of pyrazinamide (PZA).

**Methods:** PZA concentrations at random post-dose points, demographic characteristics, and clinical information were collected in a multicenter prospective TB cohort study from 18 hospitals in Korea. Data obtained from 610 TB patients were divided into training and test datasets at a 4:1 ratio. A population PK model was developed using a nonlinear mixed-effects method.

**Results:** A one-compartment model with allometric scaling for body size effect adequately described the PK of PZA. Geriatric patients with DM (age >70 years) were identified as a significant covariate, increasing the apparent clearance of PZA by 30% (geriatric patients with DM: 5.73 L/h; others: 4.50 L/h), thereby decreasing the area under the concentration–time curve from 0 to 24 h by a similar degree compared with other patients (geriatric patients with DM: 99.87 μg h/mL; others: 132.3 μg h/mL). Our model was externally evaluated using the test set and provided better predictive performance compared with the previously published model.

**Conclusion:** The established population PK model sufficiently described the PK of PZA in Korean TB patients. Our model will be useful in therapeutic drug monitoring to provide dose optimization of PZA, particularly for geriatric patients with DM and TB.

## Introduction

In the era of the coronavirus disease 19 (COVID-19), tuberculosis (TB) remains a deadly threat globally via single infection and potential coinfection with COVID-19 (Petersen, Al-Abri, Chakaya, Goletti, Parolina, Wejse, et al.). According to the WHO Global TB report in 2021, there were 10 million TB cases worldwide, with an estimated 1.5 million mortalities, representing the first mortality increase in a decade (World Health Organization, 2020). Despite remarkable TB prevention and control policies, Korea has the highest incidence rate of TB among Organisation for Economic Co-operation and Development member countries, with the number of patients imported from overseas continuing to increase (Min et al., 2021). Therefore, for successful treatment of all forms of TB, it is important to optimize the doses of currently used drugs.

The intensive phase of TB treatment uses isoniazid, rifampicin, ethambutol, and pyrazinamide (PZA) as the main components, resulting in rapid improvement of clinical symptoms (Shin and Kwon, 2015). Among the first-line anti-TB drugs, PZA has strong bactericidal activity against *Mycobacterium tuberculosis* during the early stages of treatment (Zhang and Mitchison, 2003). The area under the concentration–time curve from 0 to 24 h (AUC_0-24_) of PZA is an important predictor of early culture conversion and good bactericidal activity (Pasipanodya et al., 2013). Additionally, maximum concentration (C_max_) of PZA ranging from 20—60 μg/mL has been linked to the lower risk of treatment failure (Chideya et al., 2009; Pasipanodya et al., 2013). Considering the important role of PZA in TB treatment, it is necessary to maintain the C_max_ of the drug within this efficacy range for obtaining successful treatment of TB.

Currently, the TB treatment guidelines follow body weight-based dosing. In Korea, the recommended daily dose of PZA for treatment of drug-susceptible TB is 20–30 mg/kg, with a maximum dose of 2000 mg (Donald et al., 2012; WHO and Organization, 2017). Nonetheless, similar to other first-line anti-TB drugs, PZA also exhibits wide pharmacokinetic (PK) variability in a population (Wilkins et al., 2006; Zvada et al., 2014; Denti et al., 2015), which is challenging during patient management (Devaleenal Daniel et al., 2017). While overexposure to PZA has been shown to be strongly associated with liver damage (Younossian et al., 2005), hyperuricemia, and arthralgia (Qureshi et al., 2007), underexposure may lead to treatment failure and drug resistance (Srivastava et al., 2011).

Previous studies have shown that the PZA concentration is influenced by many factors, including genetic polymorphisms, age, comorbidities, and body weight (Graham et al., 2006; McIlleron et al., 2006; Chideya et al., 2009). Vinnard et al. (2017) reported that the clearance of PZA was 40% higher in males than in females, while Wilkins et al. (2006) reported a higher volume of distribution in males. Earlier reports also showed that the absorption of PZA was reduced in HIV patients (Gurumurthy et al., 2004; McIlleron et al., 2006; Vinnard et al., 2017). Many PK studies of PZA have been performed, most of which were in children (Thee et al., 2011; Zvada et al., 2014; Chabala et al., 2022). However, our understanding of the characteristics of PK in the elderly population is limited. It is worth noting that new TB patients aged 65 years or older accounted for 65% of the total new cases in Korea in 2020 (Cho, 2018). Elderly individuals generally have a number of physiological changes that may alter the PK of drugs, such as changes in liver and kidney function, which are responsible for the metabolism and excretion of Pyrazinamide (Mangoni and Jackson, 2004; Klotz, 2009). Furthermore, aging is closely associated with an increased risk of comorbidities, particularly type 2 diabetes mellitus (DM) (Lin et al., 2015; Park et al., 2019). DM has been widely reported to hamper successful TB treatment (Baker et al., 2011; Yoon et al., 2017). DM may reduce the exposure of PZA by increasing clearance through enhancing the activity of xanthin oxidase (XO) (Alfarisi et al., 2018), and has been linked to poor treatment outcomes (Alsultan et al., 2017). Therefore, the response should be monitored closely during treatment in geriatric patients, especially those with DM.

Therapeutic drug monitoring (TDM) is useful for optimizing drug therapy by providing a patient-tailored dose according to their PK/pharmacodynamic (PD) results (Alsultan and Peloquin, 2014; Daskapan et al., 2015). The application of TDM in clinical settings has shifted to the concept of model-informed precision dosing (MIPD) (Polasek et al., 2019; Jayanti et al., 2022). MIPD-based TDM involves the use of population PK models and prospective Bayesian forecasting to reduce the variability in treatment responses and to optimize anti-TB drug therapy. Yet, MIPD based TDM is not a common practice in high burden countries (Alffenaar et al., 2020). Several factors such as socioeconomics, healthcare infrastructure, and human resource capacity require further preparation before TDM can be fully integrated into these settings (Ghimire et al., 2016; Alffenaar et al., 2020). Additionally, population PK enables the use of sparse sampling at random post-dose points, thus avoiding the costly and laborious process of conventional TDM (Egelund et al., 2011). However, consideration should be given to the match between the characteristics of the representative population used for model development and the population in which TDM will be performed (Sturkenboom et al., 2021). Recent reports recommended the development of population PK models based on a representative population to provide appropriate dosing recommendations (Lange et al., 2020; Sturkenboom et al., 2021). Nonetheless, there have been few reports of population PK models of PZA in Asian populations, particularly in Korea. Here, we characterized and identified the influences of demographic and clinical factors on the PK of PZA by developing a population PK model, particularly for the elderly population with DM. This model could be further used to support precision therapy for TB by applying it to MIPD-based TDM.

## Materials and methods

### Ethical approval

This study was performed in accordance with the tenets of the Declaration of Helsinki and the guidelines of our institution. The current study was part of a multicenter prospective observational cohort study to develop personalized pharmacotherapy for TB patients and was conducted in 18 hospitals in Korea. We provided therapeutic drug monitoring procedure to the enrolled patients; therefore, we have registered our study in clinicaltrial.gov with the clinical trial number NCT05280886. Ethical approval was obtained from the institutional review board of each clinical site involved in the study. All patients provide written informed consent to participate in the study.

### Study data and population

Patients aged >18 years diagnosed with drug-susceptible TB and receiving a PZA-based regimen for at least 2 weeks were enrolled in the study. The enrolled patients received an oral daily dose of PZA in the range of 20–30 mg/kg and rounded to the closest tablet size as prescribed by the physician. All patients underwent sputum testing for bacteriologically confirmed diagnosis, which included the use of Xpert MTB/RIF testing capable of detecting *M. tuberculosis* and resistance to rifampin simultaneously, culture test and/or acid-fast bacilli (AFB) staining. The PZA dosing regimen followed the current Korean guidelines for TB treatment. Patients who were nonadherent or not in steady-state were excluded. The demographic characteristics of the enrolled patients, anti-TB drug treatments, comorbidities, TB diagnosis, co-medications, and laboratory testing results were collected.

### Sampling strategy of PZA

Blood samples (5 mL) were randomly collected between 0 and 24 h after the last PZA administration and were stored in heparin tubes. Typically, one sample was usually drawn from outpatients, whereas at least two samples among pre-dose and 1, 2, and 5 h after the last dose were drawn from inpatients. A portion of each 3-mL blood sample was centrifuged at 2,000 g at 4°C for 10 min to obtain plasma. The plasma was harvested within 2 h after blood sampling and stored at a temperature below −70°C until used for drug concentration measurements. The remaining 2 mL of each blood sample was stored for genotyping related to the PK of other anti-TB drugs used to treat the patients.

### Quantification of plasma PZA

The plasma concentration of PZA was measured using a validated high-performance liquid chromatography–electrospray ionization–tandem mass spectrometry method as described previously by our group (Kim et al., 2015). The previous method was modified and PZA currently used the group 2 method from the published method. Briefly, the plasma samples were prepared by protein precipitation using acetonitrile and were separated by gradient elution on a reverse-phase dC18 column. Detection was performed on the QTRAP 4000 mass spectrometer (Applied Biosystems, Foster City, CA, United States) equipped with a Turbolon-Spray source (Applied Biosystems). The calibration range was 2.0–80.0 μg/mL with a correlation coefficient of 0.9988. The lower limit of quantification (LLOQ) of PZA using this method was 2.0 μg/mL. The coefficient of variation ranges of the validation quality control samples were 5.2%–6.6% and 2.1%–5.44% for the intraday and interday precisions, respectively. The intraday and interday accuracy ranges were 93.3%–109.4% and 93.3%–109.4%, respectively.

### Population PK modeling and simulation

Population PK analysis was performed using NONMEM software (version 7.4.1; ICON Development Solutions, Ellicott City, MD, United States), and PK parameters were calculated using first-order conditional estimation via ɛ-η interaction. R software (version 4.1.0; R Development Core Team, Vienna, Austria) was used to analyze the data and generate graphs. The PZA plasma concentrations below the LLOQ were imputed to half of the LLOQ (1 μg/mL) according to Beal’s M5 method (Beal, 2001). Nonadherent patients were excluded based on the low measured concentrations of at least two anti-TB drugs used. The training and test datasets were randomly separated from the total population at a ratio of 4:1. Based on the first order absorption of the drug, the possibility of an absorption delay was examined using several different absorption models, i.e., lag-time model, sequential zero- and first-order absorption models, and transit compartment model. Interindividual variability (IIV) was assumed to follow a log-normal distribution. Additive, proportional, and combined error models were used to describe the residual error. After establishment of the base model, a correlation matrix plot was generated to identify the potential significant covariates. A likelihood ratio test was used for inclusion of covariates. Covariates were applied to the model by evaluating whether the objective function value (OFV) decreased by ≥ 3.84 with use of only one covariate in one PK parameter. Covariates were tested by forward selection and backward elimination. The most statistically significant covariates were entered first, and then other covariates with *p* < 0.01 were added sequentially.

Age, body weight, lean body weight, albumin, and total bilirubin were included as continuous covariates. Meanwhile, sex, fasting or food intake status, DM, liver disease, renal disease, and geriatric (≥60, ≥65, and ≥70 years) DM were investigated as categorical covariates of PK parameters. The effects of continuous covariates were explored using the power function with the following equation:

$${P=\left(\theta_{TV}\times \frac{{{Cont}_{COV}}_{i}}{{Cont_{COV}}_{median}}\right)}^{\theta_{p}}$$

where $\theta_{TV}$ represents the typical value of the PK parameter (*P*), ${Cont_{COV}}_{i}$ is the value of the continuous variable for the *i*th patient, ${Cont_{COV}}_{median}$ is the median value for a continuous covariate, and $\theta_{p}$ is the exponent of the power function. On the other hand, the effects of categorical variables were tested using a similar function:

$$P=\theta_{i}\times \left(1+\theta_{i+1}\times {Cat\_Cov}_{i+1}\right)$$

Following the previous equation, $\theta_{i}$ represents the estimated effect of the *i*th categorical covariate (when ${Cat\_Cov}_{i+1}$ = 0), and $\theta_{i+1}$ is the estimated effect of the *i*+1st categorical variable relative to $\theta_{i}$ (when ${Cat\_Cov}_{i+1}$ = 1). Allometric scaling of either body weight or lean body mass was applied to apparent clearance (CL/F) and central volume of distribution (Vd/F) using fixed exponents of 0.75 and 1, respectively (Anderson and Holford, 2008).

The base and final models were selected according to the decrease in the OFV generated in the likelihood ratio test, the goodness-of-fit plot, and the physiological plausibility of the estimated parameters. The final model was internally validated through prediction-corrected visual predictive check and robustness of the estimated PK, and parameters were evaluated by nonparametric bootstrap analysis. The final model was considered well validated when the mean values of the estimated parameters fell within the 95% confidence interval (CI). External validation was conducted using a test dataset that was not included in the model development. The predicted concentrations were compared with the observed concentrations using the population or the individual PK parameters estimated using the Bayesian method. The predictive performance of the final model was evaluated by comparing the model prediction errors, such as mean prediction error and absolute prediction error, with those of previously published population PK models. The criteria of previously published population PK model selection as follows: 1) similar model structure, and 2) the model established from different ethnicities compared to the study population. The external validation aims to compare and observe the final model performance with the other published models from different ethnicities when implemented in the same ethnicity with the study population. The equations used to calculate the prediction errors were as follows:

$$MPE=\frac{\sum \left(C_{Pred}-C_{Obs}\right)}{N}$$

$$APE=\frac{\sum \left(\left|C_{Pred}-C_{Obs}\right|\right)}{N}$$

## Results

### Population characteristics

A total of 613 patients were enrolled, and their plasma PZA concentration measurements were used to establish the model. Each patient contributed for one sampling point at random post dose time. The study population had a median age of 54 years (range: 19–96 years), body weight of 60.8 kg (range: 28.8–95.3 kg), and lean body weight of 48.1 kg (range: 23.1–63.79 kg), and the proportion of male patients was approximately 67%. In the total patient population, 55 patients had DM, 15 had liver disease, and 26 had renal disease (estimated glomerular filtration rate: ≤60 mL/min/1.73 m^2^). In addition, 110 patients were older than 70 years. The median body weight and lean body weight of this elderly population were 55.5 kg (range: 28.8–81.0 kg) and 44.5 kg (23.14–58.77 kg), respectively. Among the elderly population, 23 patients had DM, 11 had renal disease, and 2 had liver disease. The baseline patient characteristics are presented in Table 1.

**TABLE 1:** Patient demographic characteristics.

Characteristics	Total (*n* = 613)^a^	Training data (*n* = 488)	Test data (*n* = 125)
Sex, n (%)			
Male	415 (67.7)	325 (66.6)	90(72)
Female	198 (32.3)	163 (33.4)	35(28)
Feeding status, n (%)			
Fasted	446 (72.75)	369 (75.6	77(61.6)
Fed	167 (24.25)	119 (24.4)	48 (38.4)
DM, n (%)^b^			
Yes	84 (13.7)	55 (11.27)	29 (23.2)
No	529 (86.3)	433 (88.73)	96 (76.8)
eGFR, n (%)^c^			
Less than 60 mL/min/1.73 m^2^	36 (5.87)	19 (3.9)	17 (13.6)
More than 60 mL/min/1.73 m^2^	577 (94.13)	469 (96.1)	108 (86.4)
Regimen, n (%)^d^			
RHZE	512 (83.5)	419 (85.8)	93 (74.4)
RHZL	22 (3.6)	14 (2.9)	8 (6.4)
RZEM	21 (3.4)	16 (3.3)	5 (4.0)
RZEL	33 (5.4)	20 (4.1)	13 (10.4)
HZEL	10 (1.6)	8 (1.6)	2 (1.6)
Others	15 (2.4)	11 (2.3)	4 (3.2)
Dose, n (%)			
500 mg	4 (0.7)	3 (0.6)	1 (0.8)
1,000 mg	80 (13.1)	55 (11.3)	25 (20.0)
1,200 mg	30 (4.9)	20 (4.1)	10 (8.0)
1,250 mg	30 (4.9)	20 (4.1)	10 (8.0)
1,500 mg	386 (63.0)	328 (67.2)	58 (46.4)
1,600 mg	69 (11.3)	49 (10.0)	20 (16.0)
2000 mg	8 (1.3)	7 (1.4)	1 (0.8)
Age (y), median (range)	54.9 (19–96)	54.5 (19–96)	56.9 (19–87)
Body weight (kg), median (range)	61.03 (28.8–103.8)	60.8 (28.8–95.3)	58.81 (31.9–103.8)
Lean Body Weight (kg)			
median (range)	48.34 (23.1–69.5)	48.1 (23.1–63.79)	46.5 (27.7–69.5)
Albumin (g/dL),—3.8–5.3 g/dL	4.16 (1.6–14.1)	4.2 (2.0–14.1)	4.04 (1.6–7.6)
AST (U/L),—13–33 U/L^e^ ^,^ ^d^	29.5 (4.2–576)	27 (4.2–191)	32.6 (4.2–576)
ALT (U/L),—6–27 U/L^f^ ^,^ ^e^	22.1 (0.8–233)	19 (0.8–233)	25.3 (0.8–192)

^a^ Continuous data are given as median (range) and categorical data are given as a number (%).

^b^ DM, Diabetes Mellitus.

^c^ eGFR, Estimated Glomerular Filtration Rate.

^d^ R, rifampicin; H, isoniazid; Z, pyrazinamide; E, ethambutol; L, levofloxacin; M: moxifloxacin.

^e^ AST, Aspartate transaminase.

^f^ ALT, Alanine transaminase.

### Development of a population PK model

A one-compartment model with first-order absorption–elimination with additive residual error adequately described the PK of PZA in our population. IIV was evaluated in terms of the CL/F, Vd/F, and absorption rate constant (Ka). While IIV in both CL/F and Ka were estimated, the IIV in Vd/F was fixed to stabilize the model and obtain minimization successful. The value used to fix the IIV of Vd/F were obtained from the result of model running prior to fixing the IIV value. Allometric scaling was included for both the CL/F and Vd/F using lean body weight as a predictor of body size. The inclusion of allometric scaling with lean body weight into the base model was based on a significant reduction in OFV (∆OFV: 112.3) in comparison with using total body weight (∆OFV: 88.5). Several absorption models that were evaluated did not improve model performance and thus were not included in further analysis.

We evaluated the covariates of age, height, sex, feeding status, AST, ALT, albumin, total bilirubin, DM, advanced age (≥60, ≥65, and ≥70 years old), renal disease, and liver disease. However, none of these covariates improved the OFV. Therefore, as most TB patients in Korea are of advanced age, additional evaluation was performed using combinations of age ≥60, ≥65, and ≥70 years with comorbidities, such as DM, renal disease, and liver disease. Among these covariate groups, only age ≥60, ≥65, and ≥70 years combined with DM had a significant effect on the CL/F of PZA, in which the combination of age ≥70 years with DM showed the largest reduction of OFV by 151.3 points (*p*-value <0.001) and 2.2% decreased in IIV. Given the demographic trend of aging in the Korean population, the incorporation of a covariate representing individuals aged 70 years or older with DM would be more relevant. The CL/F value was estimated as 5.9 L/h for patients aged ≥70 years old with DM and as 4.49 L/h for other patients. The estimated values of Vd/F and Ka were 44.2 L and 1.49 h^−1^, respectively. The estimated PK parameters of PZA and the NONMEM code of final model are shown in Table 2 and Supplementary File S1.

**TABLE 2:** PZA population PK parameter estimates for the final model and bootstrap results.

Parameters^a^	Typical value (%RSE) (% η-shrinkage)^b^	Bootstrap median	Results 95% CI^c^
Fixed-effect parameters^d^			
CL/F; elder patient (≥70 years old) with DM = θ_1_ x (1+ θ_2_),θ_2_	0.32 (47.3)	0.36	0.1–0.9
CL/F (L/h); = θ_1_,θ_1_	4.49 (2.4)	4.56	4.35–4.8
Vd/F (L) = θ_3_,θ_3_	44.2 (2.7)	43.15	41.2–45.5
Ka (1/h) = θ_4_,θ_4_	1.49 (11.1)	1.41	1.14–1.84
Interindividual variability			
ω_2_; CL/F (%)	1 (60) (18)	1	0.9–1.5
ω_2_; Vd/F (%)	3 (FIX) (28.4)	—	—
ω_2_; Ka (%)	100 (1) (32.7)	1	1
Residual variability			
Additive	3.41 (14)	3.5	2.5–4.33

^a^ CL/F, apparent clearance; Vd/F, apparent volume of distribution; Ka, absorption rate constant; ω_2_, variance of interindividual variability.

^b^ % RSE, relative standard error [(SE/mean) x 100%]; % η-shrinkage, η-shrinkage = (1-SD(η)/ω) x 100%, where *η* are the between individual variation terms and ω is the population model estimate of the standard deviation in *η*.

^c^ CI, confidence interval.

^d^ Allometric scaling was applied to the CL/F and Vd/F data, and typical values reported here refer to the typical patient, with lean body weight of 48 kg.

**FIGURE 1:**
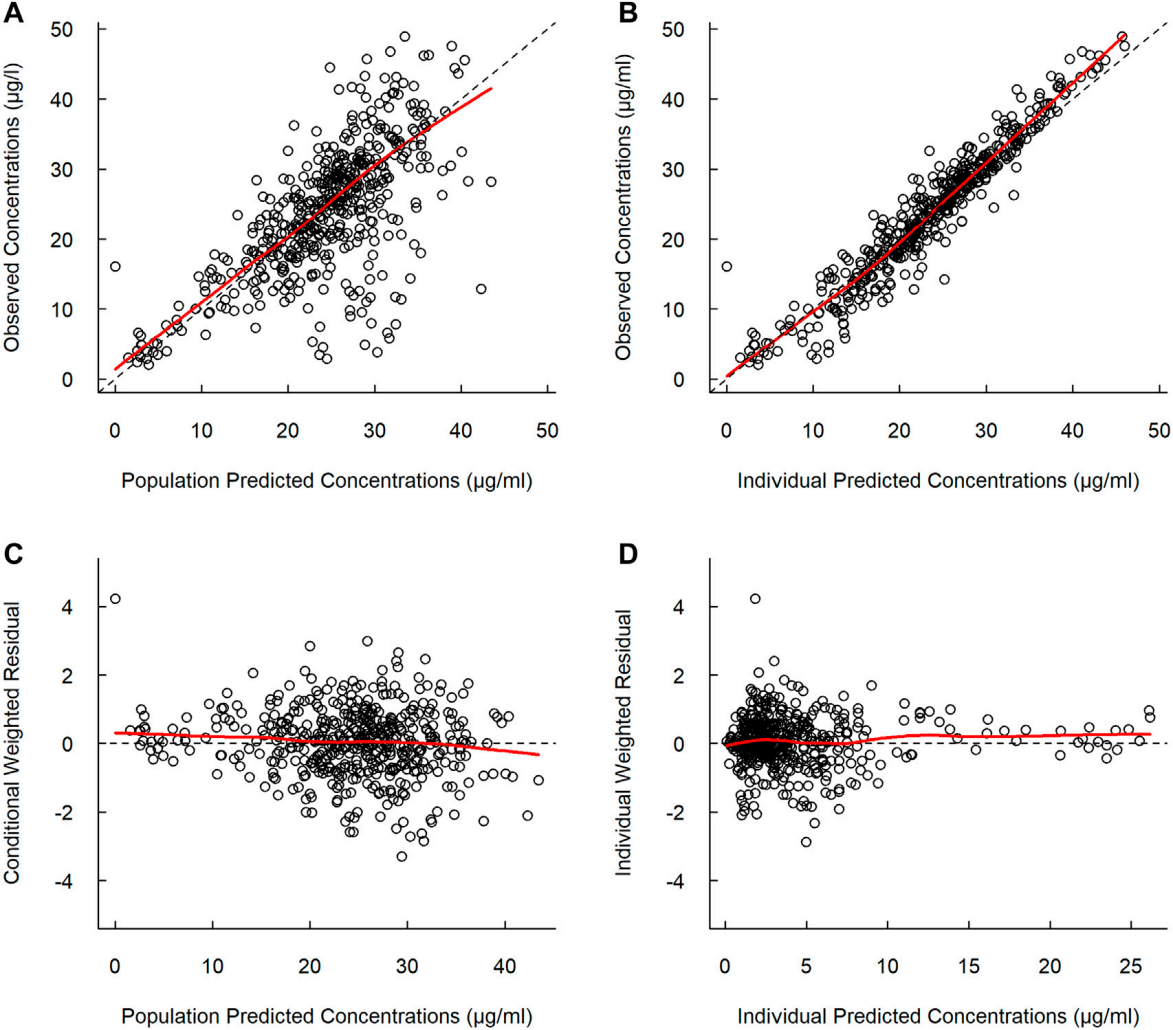
Goodness-of-fit plots of the final model. **(A)** Observed versus population predicted concentrations. **(B)** Observed versus individual predicted concentrations. **(C)** Concentration weighted residuals versus population predicted population. **(D)** Individual weighted residuals versus individual predicted concentrations. Open black circles represent the plasma concentrations of PZA, and solid red lines represent locally weighted least-squares regression according to plasma concentration.

**FIGURE 2:**
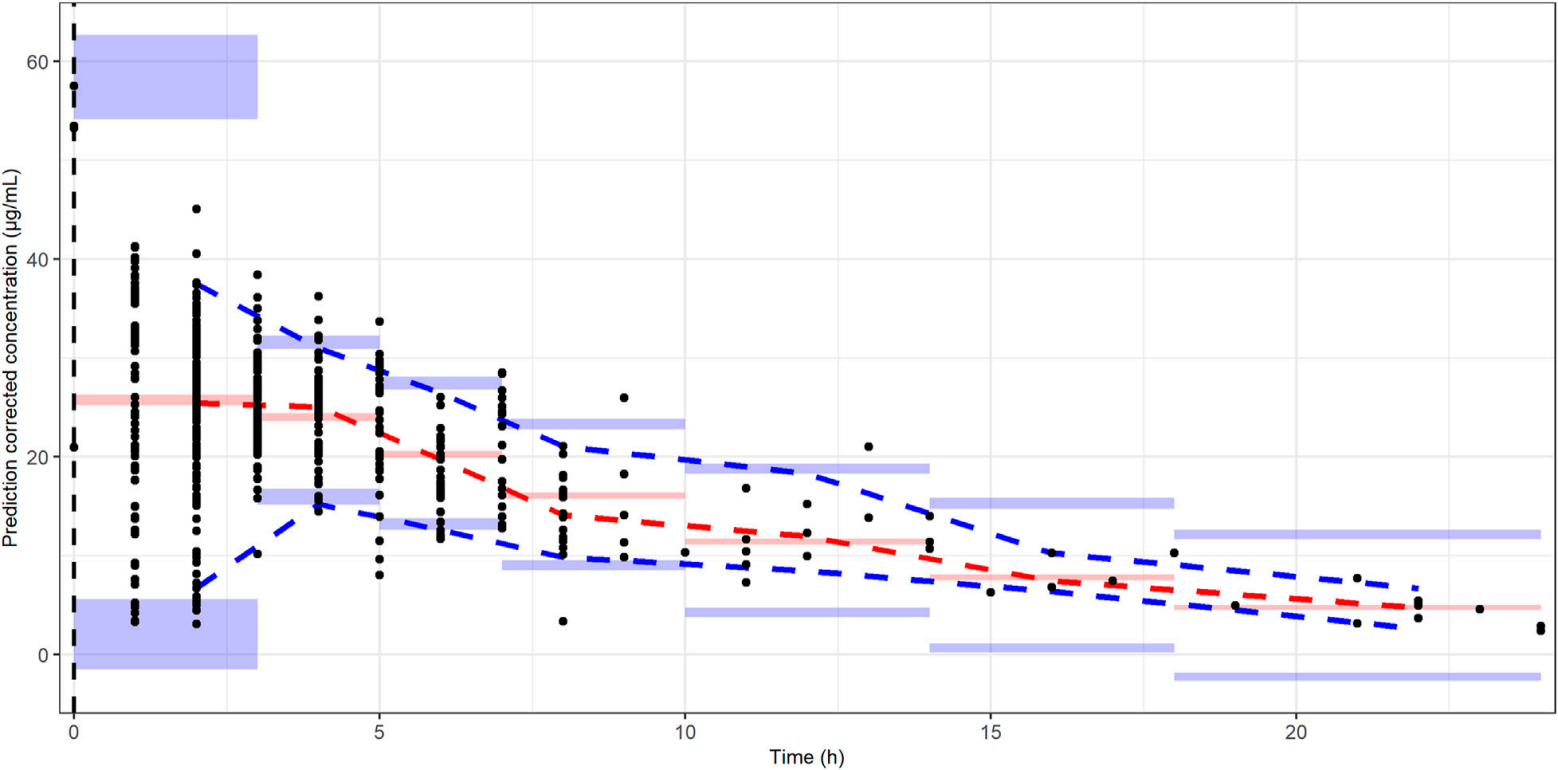
Prediction-corrected visual predictive checks. The black dots represent the observed PZA concentrations. The dash blue, red, and blue lines represent the median predicted concentration at 5th, 50th, and 95th percentiles, respectively. The shaded area represents the prediction interval with 95% confidence interval of the simulated PZA concentrations based on the final model.

The basic goodness-of-fit plots showed that the observed and predicted concentrations were evenly distributed around the line of identity without trends, and most of the predicted concentrations were distributed within two standard deviations. The structure and residual error of the model were considered appropriate without any significant bias (Figure 1). The predictive-corrected visual predictive testing also indicated a good predictive capability of the model (Figure 2). Furthermore, all other parameters showed shrinkage below 30%, indicating that the model was not overparameterized. A sparse and limited samples of PZA concentration in the absorption phase might explain the shrinkage values for Ka (>30%). In addition, parameter estimates obtained by bootstrapping analysis fell within the 95% CI and showed concordance with the results of the final model, reflecting the stability and reproducibility of the model.

### External validation of the final model

**FIGURE 3:**
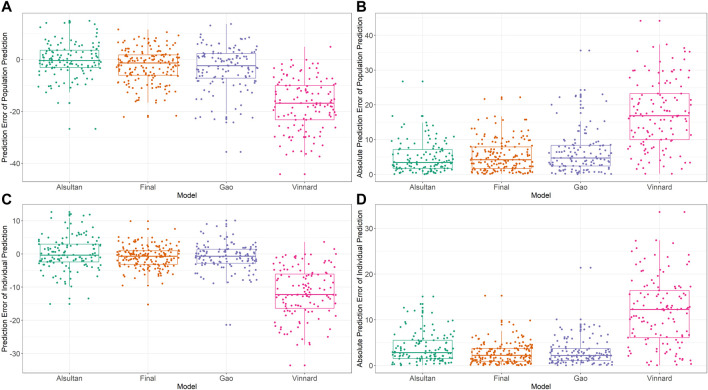
External validation and model comparison. Box and whisker plots representing prediction errors and absolute prediction errors calculated by population prediction and individual prediction in external validation. **(A)** Prediction errors computed by population prediction of the tested models. **(B)** Absolute prediction errors computed by population prediction of the tested models. **(C)** Prediction errors computed by the individual prediction of the tested models. **(D)** Absolute prediction errors computed by the individual prediction of the tested models. Different colored closed circles represent the prediction errors or absolute prediction errors for individual patients in the external validation dataset. Horizontal lines represent the medians, with the top and bottom of the boxes representing the first and third quartiles (interquartile range [IQR]), respectively, and whiskers representing extreme data within 1.5× IQR. The tested models were from Alsultan et al., 2017 (Petersen, Al-Abri, Chakaya, Goletti, Parolina, Wejse, et al.), Vinnard et al., 2017 (World Health Organization, 2020), Gao et al., 2021 (Min et al., 2021), and the final model of the present study.

External validation was conducted using the data from 125 patients from the test set. The calculated prediction error and absolute prediction error values are shown in Table 3 and visualized in Figure 3 as a comparison with other published models. Our model showed that both the prediction error and absolute prediction error were closer to zero with narrower CIs compared with the other models. These findings indicated that our model had higher accuracy and precision than those of these previous models.

**TABLE 3:** Prediction errors calculated by population prediction and individual prediction with external validation.

Model	Population prediction		Individual prediction	
	MPE^a^ (95% CI)^b^	APE^c^ (95% CI)	MPE (95% CI)	APE (95% CI)
Vinnard et al. (2017)	−17.338 (−44.175-4.87)	17.419 (0.142-44.157)	−11.678 (−33.591-3.623)	11.833 (0.024-33.591)
Gao et al. (2021)	−3.306 (−35.61-1 3.65)	6.429 (0.027-35.61)	−0.853 (−21.389-10.072)	2.978 (0.011-21.389)
Alsultan et al. (2017)	−5.507 (−22.150-14.194)	5.288 (0.011-22.156)	0.175 (−15.082-12.641)	3.845 (0.026-15.08)
Final Model	−0.253 (−26.714-14.894)	4.908 (0.028-26.741)	−0.590 (−21.389-12.641)	2.65 (0.039-15.264)

^a^ MPE, Mean Prediction Error.

^b^ CI, Confidence Interval.

^c^ APE, Absolute Prediction Error.

### Bayesian estimation of PZA PK parameters

**FIGURE 4:**
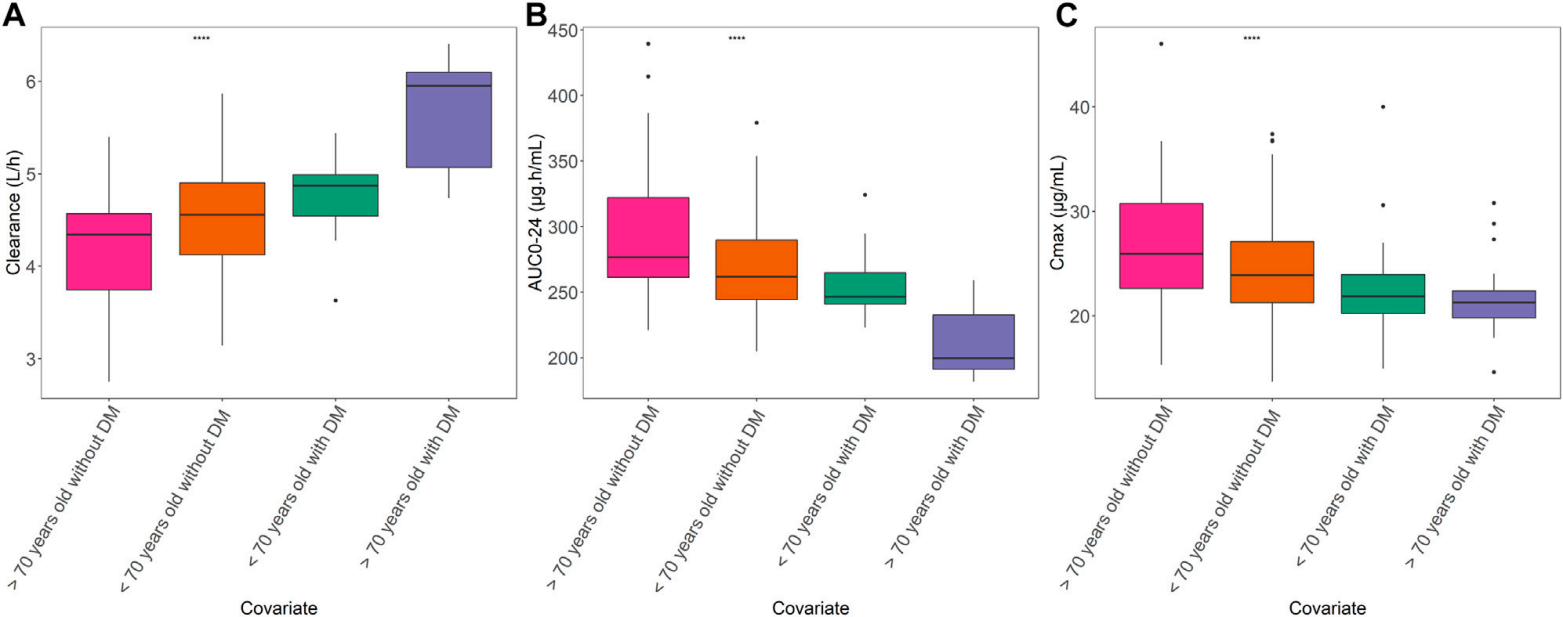
Relationships of age and DM with PK parameters. **(A)** Area under the concentration–time curve from 0 to 24 h (AUC_0-24_). **(B)** Maximum concentration (C_max_). **(C)** Apparent clearance among age groups with DM or non-DM. Box plot showing the interquartile range of each PK parameter. The groups are represented as follows: blue, >70 years with DM; red, <70 years old with DM; green, <70 years old without DM; and purple, >70 years old without DM. The straight line in the upper part of the box plot represents the ANOVA results. ****p < 0.001).

The estimated AUC_0-24_ and C_max_, normalized to a dose of 1,200 mg, according to age group and DM as comorbidities are shown in Figure 4. We divided the patients into four different groups: age <70 years with DM, age <70 years without DM, age ≥70 years with DM, and age ≥70 years without DM, with the sample size for each group was 32 patients, 344 patients, 23 patients and 87 patients, respectively. The sample size and characteristics of subgroup analysis and the concentration at 2 h post-dose among groups are presented in Supplementary Files S2, S3. Using Bayesian forecasting, the median estimated AUC_0-24_ values were 123.2 μg h/mL (interquartile range [IQR]: 120.5–132.4 μg h/mL), 131.1 μg h/mL (IQR: 122.3–131.1 μg h/mL), 99.87 μg h/mL (IQR: 95.67–116.27 μg h/mL), and 138.4 μg h/mL (IQR: 130.3–159.6 μg h/mL), respectively. The C_max_ values were 21.86 μg/mL (IQR: 20.22–23.94 μg/mL), 23.88 μg/mL (IQR: 21.26–27.11 μg/mL), 21.28 μg/mL (IQR: 19.8–22.37 μg/mL), and 25.92 μg/mL (IQR: 22.62–30.6 μg/mL), respectively. These findings indicated that the exposure to PZA in elderly patients with DM would be significantly decreased due to the higher CL/F. Furthermore, we found that DM increased the CL/F of PZA regardless of age. In addition, in the absence of DM, the CL/F of PZA tended to be decreased in the elderly population compared with younger patients. Taking both covariates into account, advanced age and the presence of DM greatly increased the CL/F of PZA. Therefore, the higher CL/F of PZA may be affected by DM as a comorbidity.

## Discussion

To our knowledge, there have been few studies regarding the interaction effect between advanced age and comorbidities in TB. As half of the new cases of TB in Korea were identified in elderly patients, there were concerns about the interaction effect of comorbidities and age that may alter the PK of anti-TB drugs, resulting in a poor treatment outcome or risk of adverse drug reactions. In this study, a PZA population PK model was developed to investigate the effects of age and other crucial clinical characteristics of Korean TB patients. Our one-compartment structural model with first-order absorption–elimination and additive residual error described the PK of PZA well. Our model was consistent with other models reported previously (Alsultan et al., 2017; Vinnard et al., 2017; Gao et al., 2021). Allometric scaling was incorporated into the CL/F and Vd/F with regard to lean body weight and improved the goodness-of-fit of the model. The CL/F of PZA estimated in this study was consistent with those of previous studies in South African TB patients reported by Rockwood et al. (2016) (CL/F: 4.17 L/h), Mugabo and Mulubwa (2019) (CL/F: 4.28 L/h), and Alsultan et al. (2017) (CL/F: 5.06 L/h).

Although direct comparison between our study and those mentioned studies were not suitable due to different body size descriptors used in the model, the incorporation of allometric scaling marked the similarity of model structure. The median total body weight and lean body weight value of our study were almost similar with Alsultan et al. (2017) and Rockwood et al. (2016), respectively. Additionally, both our study and Mugabo and Mulubwa (2019) study has high density of body weight distribution between 50—70 kg. Thus, it may possibly contribute to the concordance of CL/F estimates. Our model also showed better predictive performance compared with the model of Vinnard et al. (2017), Gao et al. (2021), and Alsultan et al. (2017) in external validation using a dataset from Korean TB patients. Thus, our results suggest that an ethnicity-specific population PK model should be utilized for TDM applications.

In addition to lean body weight, geriatric DM contributed to the IIV in the PZA concentration. We found that age, in terms of elderly patients (≥70 years old), and DM had an explanatory effect on the IIV in the CL/F of PZA in Korean TB patients using a mixed-effects model. This significant effect of advanced age with DM on the CL/F of PZA was distinguished from previous population PK studies (Otalvaro et al., 2021; Sturkenboom et al., 2021). In the subgroup analysis, we found that both younger and older patients with DM tended to have a 32% increase in CL/F compared with those without DM. Even so, the findings were statistically significant only for older patients with DM compared with the other patients. Therefore, our findings suggested an interaction between age and DM that may notably increase the CL/F of PZA. Previous studies also suggested that age may significantly affect the PK of PZA (Hagiwara et al., 2019; Kwon et al., 2020; Rousset et al., 2021). Although the identified covariate showed statistically significant, it may not be clinically important due to small decrease of IIV value.

Despite that, several studies have linked DM to a poor TB outcome and an increased risk of TB infection (Kornfeld et al., 2016; Degner et al., 2017; Kumar et al., 2017). It has been speculated that DM may reduce PZA exposure in patients (Kumar et al., 2017; Alfarisi et al., 2018). PZA is a prodrug and is metabolized to 5-hydroxypyrazinoic acid, mainly by XO, in the liver (Lacroix et al., 1989; Hussain et al., 2021). DM results in significantly elevated plasma and liver XO levels in animals with type 1 DM (Matsumoto et al., 2003), and patients with type 2 DM show increased activation of XO (Li et al., 2018; Azenabor et al., 2019). Based on these findings, we speculated that DM-induced elevation of XO may have contributed to a decrease in the PZA concentration (Alfarisi et al., 2018). Additionally, there are some evidence to suggest that DM can affect the absorption of anti-TB drugs (Nijland et al., 2006; Medellin-Garibay et al., 2015). DM may alter the expression and activity of certain transporters in the intestines that are involved in drug absorption (Dash et al., 2015), potentially leading to changes in the absorption phase of anti-TB drugs (Nawa et al., 2011). Most of the studies reported that DM alters the absorption of rifampicin (RIF) through lower gastric acid produced by hyperglycemic condition (Nijland et al., 2006), and alteration of P-glycoprotein expression and activity (Dash et al., 2015). Nonetheless, the alteration of PZA absorption phase due to DM remains uncertain. Different with RIF as the substrate of P-glycoprotein (Huerta-Garcia et al., 2019), it is worth noting that PZA is not recognized as substrates of P-glycoprotein (Alfarisi et al., 2018).

Controlling DM in elderly patients is challenging (Yakaryilmaz and Ozturk, 2017; Leung et al., 2018). The physiological changes in the elderly, characterized by insufficient insulin secretion, changes in body composition, and increased insulin resistance due to age-related sarcopenia, would increase the blood glucose level (hyperglycemia) (Yakaryilmaz and Ozturk, 2017; Mesinovic et al., 2019). This impaired glucose metabolism exacerbates the effects of DM. Therefore, elderly patients tend to have more severe effects from DM compared with the general population (Gates and Walker, 2014). Hyperglycemia activates endothelial XO, and a previous preliminary clinical study showed that blood XO was activated at high glucose concentrations (Kuppusamy et al., 2005). In fact, the hemoglobin A1c (HbA1c) concentration was reported to be significantly higher in elderly than younger subjects, as a result of the shortened life span of red blood cells due to aging (Masuch et al., 2019). The HbA1c concentration is usually used to diagnose DM in clinical settings and further reflects the blood glucose level in patients (Chehregosha et al., 2019). Earlier reports suggested that a high HbA1c concentration may increase the risk of DM complications (Sherwani et al., 2016; Lee et al., 2021). Therefore, the effects of DM on the PK of PZA may be exacerbated in elderly compared with younger patients with DM.

The effects of DM on the PK of anti-TB drugs are not well understood. Nonetheless, it was reported that the risk of treatment failure is higher in patients with TB and DM (Burhan et al., 2013; Degner et al., 2017). Even though the PK/PD target of PZA efficacy has been reported as AUC/MIC >8.42, the measurement of individual MIC was rarely determined in the clinical practice (Zheng et al., 2021). Furthermore, the extrapolation of this AUC/MIC target to the other region should be cautiously used, since the susceptibility pattern of *M. tuberculosis* may differ from region to region. Thus, the efficacy targets of AUC_0-24_ ≥ 363 μg h/mL and/or C_max_ ≥ 30 μg/mL of PZA were commonly used to adjust the dose due to its association with good treatment outcomes (Alsultan and Peloquin, 2014; Sturkenboom et al., 2021). Taking these criteria into account, none of the patients in our study population achieved the target AUC_0-24_. However, 14% of the total subjects achieved the target C_max_. As PZA is widely known for its rapid and excellent sterilizing effect, our results suggested that a higher dose of PZA is needed in DM patients, particularly in those of advanced age. It has long been suggested that a higher dose of PZA is required in TB patients, but studies have been performed mostly in younger adult TB patients (Pasipanodya and Gumbo, 2010; Alsultan et al., 2017; Chirehwa et al., 2017). As the hepatotoxicity and hyperuricemia of PZA would likely be more severe in elderly patients (Kwon et al., 2020), further controlled clinical trials, thorough evaluation of exposure of other anti-TB drugs used in the regimen, and caution with regard to dose adjustment are required to justify our suggestion. Among the few PK data related to PZA in the elderly reported to date, the results of the present study provide additional important insights into the changes in the PK of PZA in elderly patients with DM compared with other groups.

This study had several limitations. First, we collected only one sample from each outpatient in this prospective cohort study, which may have limited the precision of individual PK predictions, such as C_max_ and AUC_0-24_. However, this was a compromise to reduce the length of hospital stay of the patients. It is recommended to use a dense sampling strategy that includes at least two sampling points in order to obtain more accurate individual PK estimates. Due to our sampling strategy, the C_max_ values presented are calculated and predicted based on the developed model; therefore, have risk of incorrect estimation. Cautious interpretation of the C_max_ values should be carried out when applying it into clinical practice. Second, it was assumed that the patients took their prescription drugs on a regular basis, so the exact timing of repeat dosing was not known prior to the sampling date. Third, the effects of co-administration of other anti-TB drugs were not considered during PK evaluation. However, the interactions among anti-TB drugs remain unclear, and even if there is interaction, it most likely may be clinically unsignificant. Fourth, our population PK model and the PK characteristics described were based on Korean TB data and may vary according to ethnicity and/or patient characteristics.

## Conclusion

Using the randomized post-dose point approach, the established model adequately described the PK of PZA in Korean TB patients and showed good performance. In addition to body weight, our model identified geriatric (≥70 years) DM as an important covariate for the CL/F of PZA. We found that the geriatric DM population had a higher CL/F of PZA and lower exposure of PZA compared with other patients. The population PK model that we developed can be further used to optimize TB treatment via MIPD-based TDM implementation.

## Data Availability

The raw data supporting the conclusion of this article will be made available by the authors, without undue reservation.
