# Supplementary material for: Development of a population pharmacokinetic model of pyrazinamide to guide personalized therapy: impacts of geriatric and diabetes mellitus on clearance
Source: Front Pharmacol. 2023 May 26;14:1116226. doi: 10.3389/fphar.2023.1116226 (PMC10250603; doi:10.3389/fphar.2023.1116226)
Supplement: Supplementary file 1 [file Presentation1.pdf]

Supplementary Material

Development of a Population Pharmacokinetic Model of Pyrazinamide to Guide Personalized Therapy: Impacts of Geriatric and Diabetes Mellitus on Clearance

Ryunha Kim, Rannissa Puspita Jayanti, Hongyeul Lee, Hyun-Kuk Kim, Jiyeon Kang, I-Nae Park, Jehun Kim, Jee Youn Oh, Hyung Woo Kim, Heayon Lee, Jong-Lyul Ghim, Sangzin Ahn, Nguyen Phuoc Long, Yong-Soon Cho, Jae-Gook Shin* on behalf of the cPMTb**

*** Correspondence:**

Jae-Gook Shin, MD, PhD

E-mail: phshinjg@inje.ac.kr

**** Co-correspondence:**

Yong-Soon Cho, MD, PhD

E-mail: ysncho@gmail.com

S.1. Pyrazinamide NONMEM model code.. 2

S.2. Sample size of subgroups analysis 5

S.3. Box-whisker plot of the estimated concentration at 2 h post-dose...................................... 6

S.1. Pyrazinamide NONMEM model code

```
$PROBLEM PZA ONE COMP FINAL

$DATA pza_1_18551.csv IGNORE=@

$INPUT ID OCC TIME II SS DV AMT MDV EVID RATE=DROP CMT WEIGHT AGE
HEIGHT SEX ALB TBIL NAT2 DM  LBW OLD OLDDM

$SUBR ADVAN2 TRANS2

$PK

 OLDDM1 = 0

 IF (OLDDM.EQ.1) OLDDM1 = 1


  TVCL = THETA (1) * (LBW/48)**(THETA(6)) * (1 + OLDDM1 * THETA(8))

  TVV = THETA (2) * (LBW/48)**(THETA(7))

  TVKA = THETA (3)


  CL    = TVCL * EXP (ETA (1))

  V    = TVV * EXP (ETA (2))

  KA    = TVKA * EXP (ETA (3))


  S2    = V

  K     = CL / V

  KA   = KA


$ERROR

  IPRED = F

  W     = SQRT (THETA (4)**2 + THETA(5)**2 * IPRED**2)
```

```
  IRES = DV - IPRED

  IWRES = IRES / W

  Y     = IPRED + W * EPS (1)


$THETA

(0, 4.7)

(0, 43.1)

(0, 1.6)

(0, 3.5)

(0 FIX)

(0.75 FIX)

(1 FIX)

(0, 1)


$OMEGA

  0.2

  (0.03 FIX)

  0.2


$SIGMA 1 FIX

$ESTIMATION METHOD=1 INTER PRINT=2 FILE=pza1188COM.txt

            MSFO=1188COM.MSF

$COV PRINT=ERS

$TABLE ID OCC AMT TIME MDV EVID IPRED IWRES CRES CWRES OLDDM LBW

AGE HEIGHT WEIGHT DM
```

```
    FILE=1188COM.FIT NOPRINT ONEHEADER
$TABLE ID V CL KA K ETA(1) ETA(2) ETA(3) OLDDM AGE LBW DM
    FILE=patabtrial.pza1188COM NOPRINT FIRSTONLY ONEHEADER NOAPPEND
```

S.2. Sample size of subgroups analysis

	Young, n (%)	Old, n (%)	Total, n (%)
DM, n (%)	32 (6.6)	23 (4.7)	55 (11.2)
Non-DM, n (%)	346 (70.9)	87 (17.8)	433 (88.7)
Total, n (%)	378 (77.5)	110 (22.5)	488 (100)

DM: diabetes mellitus, Young: patients < 70 years old, Old: patients ≥ 70 years old.

S.3. Box-whisker plot of the estimated concentration at 2 h post-dose

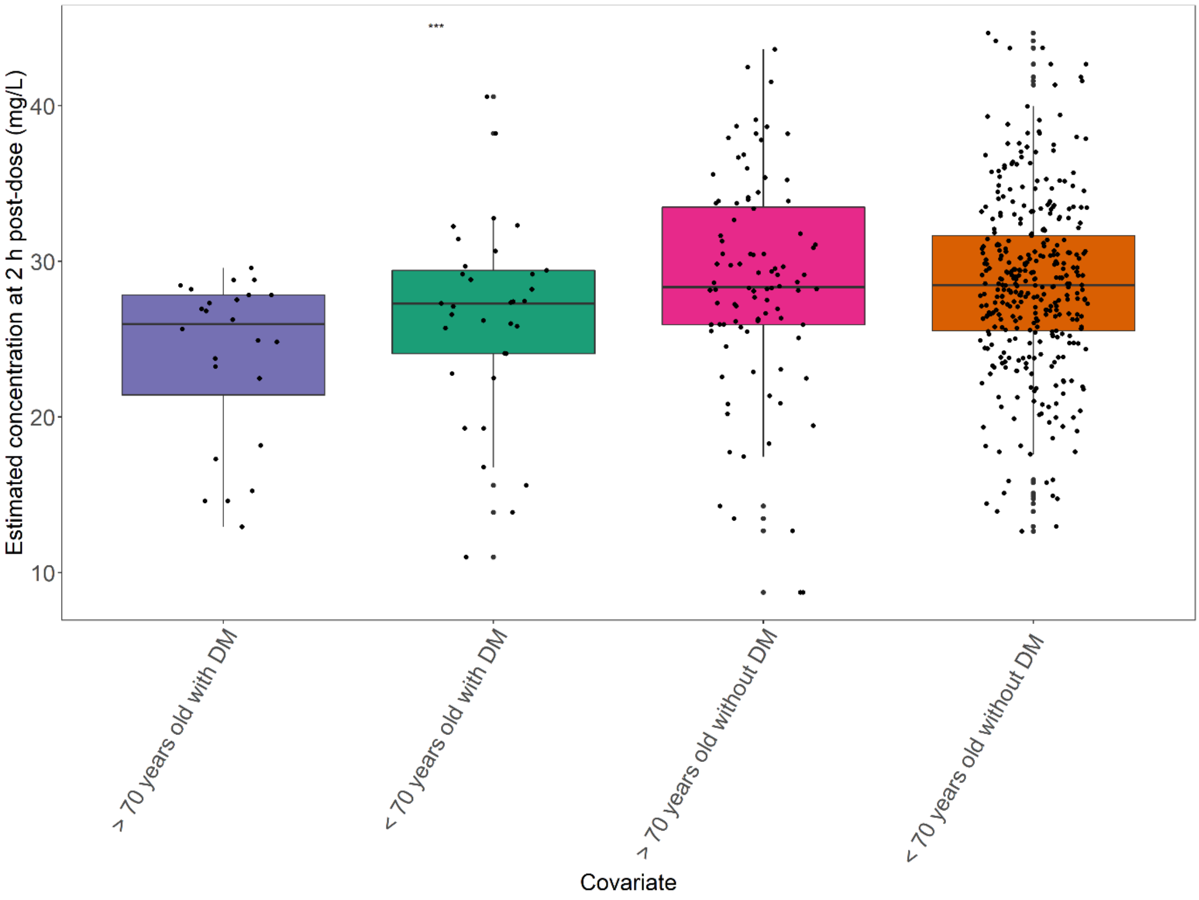

Box plot showing the interquartile range of estimated concentration at 2 h post dose among subgroups of key covariate using the final model. The groups are represented as follows: purple, > 70 years with DM; green, < 70 years old with DM; orange, < 70 years old without DM; and pink, > 70 years old without DM. The black dot represents the estimated concentration of PZA. The straight line in the upper part of the box plot represents the ANOVA results. *** $P < 0.01$).
